# PERCLOS-based technologies for detecting drowsiness: current evidence and future directions

**DOI:** 10.1093/sleepadvances/zpad006

**Published:** 2023-01-24

**Authors:** Takashi Abe

**Affiliations:** International Institute for Integrative Sleep Medicine (WPI-IIIS), University of Tsukuba, Tsukuba, Ibaraki, Japan

**Keywords:** PERCLOS, psychomotor vigilance test, vigilant attention, slow eyelid closure, sleepiness, alertness, drowsiness, sleepiness, monitoring, drowsy driving

## Abstract

Drowsiness associated with sleep loss and circadian misalignment is a risk factor for accidents and human error. The percentage of time that the eyes are more than 80% closed (PERCLOS) is one of the most validated indices used for the passive detection of drowsiness, which is increased with sleep deprivation, after partial sleep restriction, at nighttime, and by other drowsiness manipulations during vigilance tests, simulated driving, and on-road driving. However, some cases have been reported wherein PERCLOS was not affected by drowsiness manipulations, such as in moderate drowsiness conditions, in older adults, and during aviation-related tasks. Additionally, although PERCLOS is one of the most sensitive indices for detecting drowsiness-related performance impairments during the psychomotor vigilance test or behavioral maintenance of wakefulness test, no single index is currently available as an optimal marker for detecting drowsiness during driving or other real-world situations. Based on the current published evidence, this narrative review suggests that future studies should focus on: (1) standardization to minimize differences in the definition of PERCLOS between studies; (2) extensive validation using a single device that utilizes PERCLOS-based technology; (3) development and validation of technologies that integrate PERCLOS with other behavioral and/or physiological indices, because PERCLOS alone may not be sufficiently sensitive for detecting drowsiness caused by factors other than falling asleep, such as inattention or distraction; and (4) further validation studies and field trials targeting sleep disorders and trials in real-world environments. Through such studies, PERCLOS-based technology may contribute to preventing drowsiness-related accidents and human error.

**Statement of Significance**

Extensive evidence indicates that the percentage of time that the eyes are 80% closed (PERCLOS) increases with drowsiness caused by sleep loss or circadian misalignment during vigilance tests, simulated driving, and on-road driving. However, this is not always the case, such as under moderate drowsiness conditions, in older adults, and during aviation-related tasks. Although PERCLOS is one of the most accurate measures for detecting drowsiness in vigilance tests, it may not always be the most reliable indicator for detecting drowsiness while driving or in other real-world scenarios. Integrating PERCLOS with other physiological or behavioral measures may enable the robust detection of drowsiness in a variety of real-world situations and contribute to preventing drowsiness-related accidents and human error.

## Introduction

Multiple factors are involved in “sleepiness,” including sleep loss/sleep restriction [1–7], time since awakening [6–9], biological rhythms such as circadian, circasemidian, and ultradian rhythms [8, 10–12], motivation [13–15], sleep inertia [16–18], body posture [19], external stimuli such as light [20], cognitive workload [21], and orexin deficiency [22]. Nonlinear interaction between sleep homeostasis and circadian rhythm also contributes to sleepiness [6–9, 23, 24]. Sleepiness is a complex phenomenon caused by multiple factors and their interactions. In addition, elucidating the molecular basis of sleepiness remains in its early stages [25, 26]. Additionally, there is currently no single method that can adequately measure sleepiness. Although a “physiologic state of sleep need” [27] is a widely accepted definition of “sleepiness” among sleep researchers and clinicians, current methods typically measure certain aspects of self-reported, behavioral, or physiological changes associated with “sleepiness” on the basis of an operational definition of sleepiness, such as sleep propensity or drowsiness [28–34]. Sleep propensity refers to “one’s tendency to fall asleep.” [29–31] Drowsiness is a state that occurs during the transition between sleep and wakefulness and is associated with changes in cognition, behavior, vigilance, mood, motivation, autonomic and central nervous system function, other physiological states, and the self-reported experience of sleepiness [28–32]. On the basis of this definition, various methods for measuring sleepiness have been developed. Because deterioration in cognitive performance associated with sleepiness is directly related to accidents and human error [35], the evaluation of vigilance (defined here as “an ability to sustain attention to a task for a period of time” [36]), which is the performance indicator most sensitive to sleepiness [37, 38] is vital for preventing human error and accidents. The psychomotor vigilance test (PVT) is a representative method for objectively measuring vigilance relative to sleepiness [39–42]. Although the PVT and its shorter versions [42–44] are promising methods for measuring vigilance, performing these assays interrupts ongoing tasks by requiring participants to respond to stimuli that appear on a device. Therefore, methods for assessing vigilance without interfering with ongoing tasks are required.

Among approaches for passive drowsiness detection, the percentage of time that the eyes are more than 80% closed (PERCLOS) is one of the most established measures, with a large number of scientific validation studies and field trials. PERCLOS was first established in 1994 by Wierwille *et al*. for detecting driver drowsiness [45, 46]. Subsequently, a validation study of PERCLOS for detecting PVT lapses during sleep deprivation was conducted by Dinges *et al*. in 1998 [47]. The results revealed that PERCLOS was the most accurate indicator for detecting PVT lapse among several measures, including electroencephalography (EEG) and blinks [47]. Since then, PERCLOS has been extensively validated as an index for detecting drowsiness in several situations, including laboratory settings, simulated driving, and on-road driving [32, 48–50]. This narrative review discusses PERCLOS in terms of validation, comparisons with other indicators, practical applications, limitations, and future directions for further development.

## Methodology for Evaluating PERCLOS

PERCLOS can be defined as the percentage of time that the eyes are more than 80% closed [45, 47]. In previous studies of PERCLOS, the eye is defined as being closed when the eyelid is less than 20% open (0% is defined as completely closed) [45–47]. However, definitions of PERCLOS have varied greatly across studies. The calculation of PERCLOS determines eye closure based on the degree of instantaneous eyelid opening. It is possible to set eye closure to 0% or eye-opening to 0%. Previous studies have assumed complete eye closure at a given instant to be 0%, with (1) 100% representing eyes wide open and 0% representing eyes closed [45]; (2) the percentage of the distance between eyelids when the diameter of the iris is represented as 100% [47, 51]; (3) the percentage of the pupil occluded by the eyelid with the diameter of the pupil was 100% [52]. Eye-opening of less than 25% [53] or 30% [47, 54–57] has also been used as a definition of eye closure in some previous studies. In some cases, to obtain a measure of PERCLOS in a simplified manner, the eye is considered to be closed (4) when the pupil cannot be detected [58, 59], or (5) the eye aspect ratio [60] is below a definite value [61]. Some studies exclude fast blinks as eye closure [47, 51, 52, 62], while others include this measure [58, 63]. The definitions of fast eyelid closure include <250 ms [47, 64], <400 ms [52, 62], or <500 ms [51]. The sampling frequencies for recording the degrees of eyelid opening include 2 Hz [65], 3 Hz [66], 6 Hz [56], 10 Hz [51, 67], 24 Hz [54], 60 Hz [68, 69], or 120 Hz [52, 55]. A sufficient sampling rate is required when fast blinks are excluded. The analysis may be performed manually on the basis of the video recording of the eyes [47, 51, 65] or automatically by a device [70–72]. In the case of manual analysis, it takes a substantial amount of time to evaluate each frame. However, automatic measurements may cause discrepancies in values even when PERCLOS values are simultaneously measured using several instruments [71]. The effects of these differences in definitions and instruments on the accuracy of vigilance detection require further study. Initially, the percentage of time with eyes closed (%TEC) measured by infrared reflectance oculography (i.e. Optalert Drowsiness Measurement System, Sleep Diagnostics Pty Ltd, Melbourne, Australia) was not referred to as PERCLOS [73, 74]. However, a recent study reported %TEC as a measure of PERCLOS [75]. The %TEC is defined as the “percentage of time that the eyes are deemed closed in each minute.” [74] This review also discusses %TEC.

## What Activities Does PERCLOS Reflect?

PERCLOS can be used to detect decreased vigilance in vigilance tasks using both visual and auditory stimuli [76]. Thus, the association between PERCLOS and reduced vigilance is not merely caused by the blockage of the acquisition of external visual information by closing the eyes, but also by reduced activity of the central nervous system. Neural correlates of slow eyelid closure, a component of PERCLOS, during sleep deprivation have been reported using functional magnetic resonance imaging, which indicated reduced coupling within the default mode network (DMN) and weak anticorrelations between the DMN and the dorsal attention network (DAN) [77, 78]. Furthermore, reduced connectivity within the DMN and anticorrelation between the DMN and DAN were reported to predict intraindividual temporal fluctuations in response speed during the auditory vigilance task [78]. Similar reductions have been reported under sleep-deprived conditions during a visual attention task [79]. Thus, such brain activity is likely to be responsible for the occurrence of slow eyelid closure and the associated reduction in vigilant attention.

## Use of PERCLOS Under Fixation, PVT, and Oxford Sleep Resistance Test

Several studies using tasks that do not necessarily require gaze shifts have repeatedly demonstrated that PERCLOS shows an increase that is related to drowsiness manipulations (Table 1), such as sleep deprivation or partial sleep restriction when conducting the PVT [47, 51, 52, 62] or the Oxford Sleep Resistance (OSLER) test [58]. The latter is a behavioral test that is similar to the maintenance of wakefulness test (MWT), which requires a response to a light stimulus occurring every 3 s for 40 min [80]. A similar index measured using Optalert, %TEC, has also been shown to increase with drowsiness manipulation when looking ahead [73] or when conducting PVT [70, 74, 81, 82]. Additionally, the measurement of PERCLOS during a simple reaction task in the field has captured the disturbance of circadian rhythms among two types of air traffic controllers: those who experience fewer night shifts and those who experience a higher frequency of night shifts [54]. It should be noted that not all studies have shown an increase in PERCLOS with PVT measurement during sleep deprivation [64, 70].

**Table 1.** Summary of the effects of drowsiness manipulation on PERCLOS

Reference	Device	Task	Participants (*N*, type, and age range)	Drowsiness manipulation	Results
Fixation, PVT, OSLER test					
Dinges *et al*. [47]	CCTV Camera	PVT	*N* = 10; healthy adults; 21–35 years	42 h of sleep deprivation	Changes corresponded to increased PVT lapses in the 42-hour period
Abe *et al*. [58]	EMR-9^*^	OSLER test	*N* = 9; healthy adults; 19–30 years	One night of partial sleep deprivation (4 h sleep)	Changes corresponded to increased missed responses after partial sleep deprivation
McKinley *et al*. [64]	EC6^†^	PVT	*N* = 10; healthy adults; 18–42 years	Experimental testing began after 14 h awake and continued until 28 h of sleep deprivation	No change
Chua *et al*. [52]	ISCAN eye tracker^‡^	PVT	*N* = 24; healthy adults; 25.9 ± 2.8 years	40 h of sleep deprivation	Changes corresponded to the profile of PVT performance
Anderson *et al*. [73]	Optalert^§^	Looking ahead	*N* = 28; healthy adults; 18–34 years	30 h of sleep deprivation	Increased after 26 h of wakefulness compared with the first 16 h of wakefulness
Ftouni *et al*. [74]	Optalert^§^	Auditory PVT	*N* = 10; healthy adults; 20–25 years	40 h of sleep deprivation	Increased at 24 to 25 h of wakefulness compared with the first 16 h after awakening.
Chua *et al*. [62]	ISCAN eye tracker^‡^	PVT	*N* = 12; healthy adults; 22–30 years	26 h of sleep deprivation	Increased after usual bedtime
Ftouni *et al*. [82]	Optalert^§^	Auditory PVT	*N* = 22; night shift workers; 33.4 ± 11.8 years	Tested groups (1) within (*N* = 14) or (2) outside (N = 8) the acrophase (± 3 h from the peak) of 6-sulphatoxymelatonin (aMT6s)	Increased in group (1) compared with group (2)
Jackson *et al*. [70]	Copilot [66]	PVT	*N* = 22; healthy adults; 18–26 years	(1) One night of sleep deprivation; (2) a normal night of sleep	No change
Jackson *et al*. [70]	Optalert^§^	PVT	*N* = 22; healthy adults; 18–26 years	(1) One night of sleep deprivation; (2) a normal night of sleep	Increased in condition (1) compared with condition (2)
Abe *et al*. [51]	EMR-9^*^	PVT	*N* = 16; healthy adults; 20–49 years	38 h of sleep deprivation	Increased after 18 h of awake, peaked at 22 h
Wilkinson *et al*. [81]	Optalert ^d^	PVT	*N* = 15; healthy adults; 37.6 ± 11.6 years	(1) Benzodiazepine administration; (2) placebo	Increased after (1) compared with (2)
Zhang *et al*. [54]	A front-facing laptop camera	A simple reaction task	*N* = 32; air traffic controllers at terminal control unit; 23–36 years	Four shift zones: (1) 8:00–12:00; (2) 12:00–18:00; (3) 18:00–24:00; (4) 24:00–8:00	Increased in shifts (3) and (4) compared with shifts (1) and (2)
Zhang *et al*. [54]	A front-facing laptop camera	A simple reaction task	*N* = 35; air traffic controllers at air control unit; 23–38 years	Four shift zones: (1) 8:00–12:00; (2) 12:00–18:00; (3) 18:00–24:00; (4) 24:00–8:00	No difference
Simulated driving					
Dingus *et al*. [100]	Unobtrusive camera/monitor system	Fixed based; on the nighttime interstate driving	*N* = 6; drivers; over 21 years	(1) Driving 19 h after participants’ normal wake-up time; (2) driving in the early evening after a normal night’s sleep	Changes corresponded to the lane position measures
Mortazavi *et al*. [89]	Head-mounted eye tracking system	Fixed-based; monotonous highway driving	*N* = 13; professional truck drivers; 23–55 years	(1) A night session after 18–19 h of wakefulness; (2) morning session after at least 8 h of sleep	Increased in the night session (1) compared with the morning session (2)
Golz *et al*. [71]	Three anonymous devices	Fixed-based; monotonous motorway driving	*N* = 16; healthy adults; 19–29 years	Overnight driving sessions from 11:30 pm To 8:30 am	Corresponded changes with KSS and SDL progressively increased throughout the night and peaked at 6 am.
Merat and Jamson [53]	FaceLAB^‖^	High fidelity; motorway driving	*N* = 17; shift workers; 31.4 ± 5.4 years	(1) After a night shift; (2) after a normal night’s sleep	Increased in (1) compared with (2)
Merat and Jamson [53]	FaceLAB^‖^	High fidelity; motorway driving	*N* = 16; older adults; 53.2 ± 5.5 years	(1) Afternoon after consuming a large lunch; (2) baseline trial during the morning	Increased in (1) compared with (2)
Alvaro *et al*. [65]	Digital video recordings using infrared light	Fixed-based; monotonous nighttime highway driving	*N* = 20; professional drivers; 41.9 ± 8.3 years	24 h of sleep deprivation	Increased during sleep deprivation
Jackson *et al*. [70]	Copilot [66]	Fixed-based; monotonous nighttime highway driving	*N* = 22; healthy adults; 18–26 years	(1) One night of sleep deprivation; (2) after a normal night of sleep	No change
Jackson *et al*. [70]	Optalert^§^	Fixed-based; monotonous nighttime highway driving	*N* = 22; healthy adults; 18–26 years	(1) One night of sleep deprivation; (2) after a normal night of sleep	Increased in (1) compared with (2)
Jackson *et al*. [67]	Copilot [66]	Fixed-based; monotonous nighttime highway driving	*N* = 12; healthy professional drivers; 23–62 years	(1) After 24 h of sleep deprivation; (2) after a normal night of sleep	Increased in (1) compared with (2)
Wang and Xu [92]	SmartEye Pro^¶^	High-fidelity motion-based highway daytime driving	*N* = 16; Night shift workers; 24–40 years	Driving after having worked an 8 h night shift	Increased with the KSS-measured drowsiness levels
Caponecchia and Williamson [69]	SmartEye Pro^¶^	Fixed based; arterial road and highway driving	*N* = 41; healthy adults; Group 1: 37.0 ± 8.3 years; Group 2: 42.1 ± 6.3 years; Group 3: 38.4 ± 5.9 years	Different levels of sleep deprivation (Group 1: no sleep deprivation; Group 2: 2 h deprivation; Group 3: 4 h deprivation) and time of day (morning and evening)	No change
Puspasari *et al*. [59]	EyeLink II^g^	Fixed based; highway driving	*N* = 13; healthy adults; 30.2 ± 5.0 years	Sleep duration was divided into two levels: (1) 4 h or (2) 8 h	Increased in (1) compared with (2)
Wilkinson *et al*. [81]	Optalert^#^	Fixed-based; monotonous nighttime highway driving	*N* = 15; healthy adults; 37.6 ± 11.6 years	(1) Benzodiazepine administration; (2) placebo	Increased in (1) compared with (2)
Wörle *et al*. [90]	SmartEye Pro^¶^	High fidelity; monotonous manual driving after take-over from automated driving	*N* = 61; regular drivers; 38.1 ± 11.9 years	KSS determined “alert” (KSS ≤ 4) or “sleepy” (KSS ≥ 7); EEG defined “after sleep”	Increased after sleep relative to the alert state.
Murata *et al*. [57]	Drowsimeter R100^**^	Fixed-based; driving along a straight road with following a leading car	*N* = 13; healthy adults; 21–25 years	(1) Drowsy state: one night of staying up all night; (2) aroused state: sufficient sleep	Increased in (1) compared with (2)
Arefnezhad *et al*. [91]	N/A	Fixed based with four bass shakers for generating the vibration in the car; motorway automated and manual driving	*N* = 89; drivers; 20–85 years	(1) Fatigued conditions (16 h awake or 4 h sleep the previous night); (2) rested condition	Differences between the automated and manual modes were greater in (1) compared with (2)
On-road driving					
Ftouni *et al*. [74]	Optalert^§^	Participants’ commute to or from work	*N* = 27; rotating and permanent night shift-working nurses; 41.6 ± 12.5 years	Driving to and from night shifts	Increased after the night shift compared with that before the night shift
Lee *et al*. [95]	Optalert^§^	2-h daytime driving on a closed driving track	*N* = 16; night shift workers; 18–65 years	(1) Post-night shift driving following night shift work; (2) driving after a night of at least 5 h of sleep with no night shift work	No change
Ahlström *et al*. [94]	Smart Eye System^¶^	Automated driving of a 90-km section on a dual-lane motorway with real traffic	*N* = 89; healthy drivers	(1) Nighttime (1:00 or 3:00); (2) daytime (15:00 or 17:00)	Increased at nighttime
Cai *et al*. [72]	Seeing Machines’ DMS^‖^	2 h driving around a closed driving track	*N* = 16; young adults; 21–33 years	(1) After 29 h of sleep deprivation; (2) after 8 h sleep	Increased after deprivation
Cai *et al*. [72]	Seeing Machines’ DMS^‖^	2 h driving around a closed driving track	*N* = 17; older adults: 50–65 years	(1) After 29 h of sleep deprivation; (2) after 8 h sleep	No change
Cori *et al*. [99]	Optalert^§^	A week of regular naturalistic driving	*N* = 30; Obstructive sleep apnea (*N* = 15); 46.5 ± 7.3 years; healthy controls (*N* = 15); 48.7 ± 7.2 years	Patients with obstructive sleep apnea or healthy controls	No difference
Aviation-related task					
McKinley *et al*. [64]	EC6^†^	Target acquisition task	*N* = 10; healthy adults; 18–42 years	Experimental testing began after 14 h of wakefulness and continued until 28 h of sleep deprivation	No change
McKinley *et al*. [64]	EC6^†^	Unmanned aerial vehicle task	*N* = 10; healthy adults; 18–42 years	Experimental testing began after 14 h of wakefulness and continued until 28 h of sleep deprivation	No change

Optalert measured %TEC (percentage of time with eyes closed); CCTV, closed-circuit television; DMS, driver monitoring system; KSS, Karolinska Sleepiness Scale; OSLER, Oxford Sleep Resistance; PVT, Psychomotor Vigilance Test; SDL, standard deviation of lateral position in lane.

^*^nac Image Technology Inc., Tokyo, Japan.

^†^EyeCom, Inc., Reno, Nevada.

^‡^ISCAN, Inc., Woburn, MA.

^§^Sleep Diagnostics Pty Ltd., Melbourne, Australia.

^‖^Seeing Machines, Canberra, Australia.

^¶^Smart Eye AB, Gothenburg, Sweden.

^#^SR-Research, Osgoode, Ontario, Canada.

^**^Phasya S.A., Seraing, Belgium.

The accuracy of PERCLOS in detecting PVT lapse and nonresponse (response time [RT] ≥3 s) during the OSLER test associated with drowsiness manipulation is shown to be higher than various measures, including slow eye movements, blink parameters, pupil diameter, heart rate variability, and EEG-based measures as demonstrated in Table 2 [47, 52, 58]. While one study reported that %TEC had stronger correlations with PVT performance than that of PERCLOS measured using Copilot or Johns Drowsiness Scale (JDS: a composite drowsiness measure of multiple eye metrics using the Optalert [83]) [70], high accuracy in detecting performance impairment during the PVT and OSLER test is not always achieved in the %TEC measured with Optalert [73, 74, 81, 84]. The cause of this discrepancy between %TEC and PERCLOS is unclear; however, the definition of eye closure or the measurement device may affect the difference in accuracy between these measures.

**Table 2.** Summary of comparisons of accuracy between PERCLOS and other indicators in drowsiness detection

Reference	Drowsiness manipulation	Calculation method for accuracy	The best index for identifying drowsiness events
Fixation, PVT, OSLER test			
Dinges *et al*. [47]	42 h of sleep deprivation	Pearson’s or Spearman’s rank correlations: (1) PVT lapse frequency, and (2) cumulative lapse duration time	(1–2) PERCLOS
Abe *et al*. [58]	One night of partial sleep deprivation (4 h sleep)	AUC for discriminating (1) between the EP0 (no missed response) epoch and EP1–6 (at least one missed response) epoch, and (2) between the EP0–2 (two consecutive missed responses) epoch and EP3–6 (three consecutive missed responses) epoch.	(1–2) PERCLOS
Chua *et al*. [52]	40 h of sleep deprivation	(1) Pearson’s correlation analysis; (2) AUC to identify a threshold increase (>25%, >50%, or >75%) relative to baseline in PVT lapses.	(1–2) PERCLOS^*^
Anderson *et al*. 73^†^	30 h of sleep deprivation	AUC to identify a threshold increase ((1) > 50%, (2) > 75%) relative to baseline in PVT lapses (recordings were obtained while participants looked directly ahead), and (3) AUC to identify KSS 6 or above	(1–3) JDS
Ftouni *et al*. [74]	40 h of sleep deprivation	AUC to identify a threshold increase in auditory PVT lapses ((1) > 25%, (2) > 50%, and (3) > 75%) relative to the first 16 h of wakefulness	(1–3) PosAVR
Wilkinson *et al*. 84,^‡^	8 h sleep or one night of 4 h sleep restriction	AUC of missed signals: (1) ≥ 4 consecutive missed signals; (2) ≥ 4 total missed signals in the OSLER test	(1–2) IED
Chua *et al*. [62]	26 h of sleep deprivation	Intraclass correlation coefficient to assess the reproducibility of individual differences between two visits of 26 h of sustained wakefulness during PVT	PERCLOS
Jackson *et al*. [70]	One night of sleep deprivation or after a normal night of sleep	Spearman’s rank correlation: (1) median RT, and (2) lapse during PVT	(1–2) %TEC
Wilkinson *et al*. [81]	Benzodiazepine administration or placebo	(1) AUC for detecting one or more PVT lapses, and (2) AUC for detecting two or more PVT lapses in a 1-min bin	(1) JDS and (2) %TEC in the placebo condition; (1) JDS, and (2) IED in the BZ condition
Abe *et al*. [51]	38 h of sleep deprivation	Intraclass correlation coefficients between probabilities of PVT response (RT ≥ 300 ms) and a new PERCLOS-based algorithm (PVT-E) or PERCLOS	PVT-E
Simulated driving			
Golz *et al*. [71]	Overnight driving sessions from 11:30 PM to 8:30 AM	Test error of nonlinear discrimination analysis for (1) KSS, and (2) standard deviation of lateral position in the lane	(1–2) EEG/EOG-based classifier
McDonald *et al*. 101,^§^	Different times of day: daytime, early evening, and late in the evening (and early morning).	Accuracy and AUC for detecting drowsiness-related lane departures	Random forest steering algorithm
Jackson *et al*. [70]	One night of sleep deprivation or after a normal night of sleep	Spearman’s rank correlation: (1) standard deviation of lateral position, and (2) number of crashes	(1) JDS; (2) %TEC
McDonald *et al*. 68,^‖^	Driving during the daytime; driving during the late evening and early morning.	AUC for detecting drowsy-related lane departures	MLC_DMN
Puspasari *et al*. [59]	Sleep duration was divided into two levels: 8 h or 4 h	Spearman’s rank correlation: (1) KSS, (2) line crossing, and; (3) incident frequency	(1) Blink duration; (2) microsleep; (3) saccadic peak velocity
Puspasari *et al*. [59]	Sleep duration was divided into two levels: 8 h or 4 h	AUC for discriminating: (1) alert and low-level fatigue conditions, and (2) low-level and heavy fatigue conditions (Alert: KSS 1–5; low-level fatigue: KSS 6–7; heavy fatigue: KSS 8–9 and experienced line crossing at least once)	(1–2) Blink duration
Wilkinson *et al*. [81]	Benzodiazepine administration or placebo	Sensitivity and specificity for detecting driving simulator crashes	Blink duration
Aviation-related task			
McKinley *et al*. [64]	Experimental testing began after 14 h awake and continued until 28 h of sleep deprivation	Partial correlations of reaction time to detect pop-up enemy targets while simulated flying during target acquisition task	Approximate entropy of pupil position
Sleep disorders			
Kratzel *et al*. 55,^¶^	MWT in persons with sleep disorders	AUC for discriminating PSG-measured sleep in an MWT trial	PERCLOS

See Table 1 for information about devices and participants; information on studies not included in Table 1 is given in the footnotes.

AUC, area under the receiver operating characteristic curve; BZ, benzodiazepine; EEG: electroencephalography; EOG, electrooculography; EP, error profile; IED, inter-event duration; JDS, Johns Drowsiness Scale; MLC_DBN: dynamic Bayesian network (DBN) that integrates multiple random forest observations from steering angle and pedal input with maneuver-level context from vehicle speed and acceleration. MWT, maintenance of wakefulness test; %TEC, percentage of time with eyes closed; PosAVR, positive amplitude-velocity ratio of each blink; PSG, polysomnography; PVT, psychomotor vigilance test; PVT-E, eye-metrical estimation version of the PVT.

^*^Note that the AUC of RR-interval PSD (0.02–0.08 Hz) was not significantly different from that of PERCLOS for the 25% and 75% PVT lapse increases.

^†^Recordings were during looking ahead.

^‡^Device: Optalert (Sleep Diagnostics Pty Ltd., Melbourne, Australia); *N* = 33; participant type: healthy adults; age range: 18–70 years.

^§^Device: NA; *N* = 72; participant type: healthy drivers; age range: 21–68 years.

^‖^Device: FaceLab 5.0 (Seeing Machines, Canberra, Australia); *N* = 7 for test dataset and *N* = 65 for training data; participant type: healthy adults: age range: 22–57 years (test dataset).

^¶^Device: Drowsimeter R100 (Phasya S.A., Seraing, Belgium); *N* = 30; participant type: suspected or diagnosed narcolepsy, idiopathic hypersomnia or obstructive sleep apnea; age range: 18–80 years.

Importantly, PERCLOS values during PVT have been shown to be similar in repeated exposures to sleep deprivation, indicating that the PERCLOS response to sleep deprivation is trait-like [62]. This is similar to the PVT performance [85–87]. In addition, PERCLOS has been found to exhibit a higher intraclass correlation coefficient (ICC) compared to various measures, including several spectral bands of EEG power, heart rate, HRV, blink rate, self-reported measures, and PVT measures (Table 2) [62]. The lower intra-individual variation of PERCLOS under the same conditions suggests that PERCLOS may be more sensitive to changes in sleepiness compared to other indices in the PVT.

A method for detecting vigilance deterioration in PVT with greater accuracy than PERCLOS has been reported. A PERCLOS-based algorithm that integrates multiple eye metrics was found to outperform PERCLOS, with ICCs of PVT RT ≥ 300 ms [51]. However, this algorithm requires the use of electrooculography (EOG) and high-precision eye cameras. Implementing this algorithm with unobtrusive devices is needed for practical use. In addition, future research should investigate whether this method is effective in detecting drowsiness-related events in both simulated and real-world settings, on top of vigilance tests.

## Use of PERCLOS Under Various Driving Conditions

Validation of PERCLOS as a measure of drowsiness in operational environments requires both simulated and field studies [88]. Many validation studies for PERCLOS have been conducted, particularly in the context of driving, with numerous simulation studies reporting increases in PERCLOS or %TEC during driving after sleep deprivation [57, 67, 70] or partial sleep deprivation [59], overnight driving [65, 71, 89], driving after a night shift [53], driving at post-lunch dip [53], driving during sleep inertia [90], driving after benzodiazepine administration [81], or automated driving (combined operation of adaptive cruise control and lane-keeping assistance) when compared to manual driving [91]. The effect of automated driving on PERLOS is more pronounced under fatigued conditions (extended wakefulness or sleep deprivation) [91]; PERCLOS has been shown to increase with self-reported sleepiness during simulated driving [57, 90, 92]. On-road driving studies have also reported increases in PERCLOS during manual driving with sleep deprivation in younger adults [72], during nurses’ commutes after a night shift compared to before a night shift [93], and during motorway driving at night time [94].

There are several issues that need to be addressed to increase the efficacy of PERCLOS in detecting drowsiness during on-road driving. First, there are conflicting pieces of evidence regarding the effectiveness of PERCLOS or %TEC in detecting drowsiness after night shifts. Some studies have found that PERCLOS or %TEC increases after a night shift when driving on a simulated course [53] or during an on-road commute [93]. However, other research found no changes in %TEC during daytime driving on a closed driving track after working a night shift when compared to a night without working a night shift [95]. Further development and validation of drowsiness monitoring are urgently needed to reduce the higher risk of accidents during driving after a night shift [95–97]. Second, it is necessary to determine the extent to which PERCLOS can detect moderate drowsiness during on-road driving. PERCLOS in simulated driving was not sensitive to moderate sleep restriction (2–4 h of sleep reduction) [69]. In addition, the relationship between PERCLOS and SD of the lateral lane position is low when the drivers are in mild and mid-range fatigue conditions [71]. However, there are no studies investigating the effect of moderate sleep restriction on PERCLOS during on-road driving. Thus, future research should examine the extent to which sleep restriction affects PERCLOS during on-road driving. Third, there may be limitations to using PERCLOS for detecting drowsiness in older adults. Studies have shown that PERCLOS in older adults does not always correspond to underload in automated simulated driving compared to manual driving [91] or deterioration in on-road performance due to sleep deprivation [72]. In the latter study, older drivers showed increased lane deviation as a result of sleep deprivation, suggesting that factors other than falling asleep, such as inattention and distraction related to drowsiness [98], may contribute to impairments in driving performance due to drowsiness. Developing methods to detect drowsiness-related inattention or distraction may help to improve the low sensitivity of PERCLOS to drowsiness in older adults. Fourth, there are limited hours in which PERCLOS is more sensitive to drowsiness during on-road driving. A simulated driving study showed a higher incidence of PERCLOS in automated driving compared to manual driving during daytime [91]. However, this effect was not observed during on-road driving in the daytime [94]. Safety-sensitive real-world driving conditions may reduce the occurrence of drowsiness due to the underload of the automated driving mode. Further identification of which populations and situations are more likely to exhibit increased PERCLOS associated with automated driving will be important to improve the utility of PERCLOS in the upcoming need for monitoring driver’s states during automation mode. Fifth, there are still few studies on the utility of PERCLOS while driving in patients with sleep disorders. One study measured %TEC during on-road naturalistic driving in the obstructive sleep apnea group and found no difference in %TEC compared to healthy controls [99]. More research targeting sleep disorders provides a better understanding of whether eye metrics, including PERCLOS, are appropriate for assessing fitness-to-drive [99].

What behavioral parameters are related to PERCLOS during drowsy driving? There is generally robust evidence of a relationship between PERCLOS and measures of lateral position variability (e.g. number of lane departures or SD of lateral position) during simulator driving [59, 65, 71, 89, 100], with the exception of one study [70]. However, this correlation tends to decrease in low drowsiness conditions [71]. Studies investigating the relationship between PERCLOS and crashes have produced conflicting results, with some studies finding a correlation [59, 65, 70] and others finding no correlation [67, 70, 81]. There are fewer studies that have used braking reaction time as an indicator, and these studies have also yielded inconsistent results, with some reporting a correlation [65] and others, not [67].

Is PERCLOS more sensitive than other indicators in detecting drowsiness during driving? In vigilance tests, PERCLOS has consistently been shown to be more accurate in detecting drowsiness than other indicators; however, this is not always the case in the driving situation as shown in Table 2 [59, 68, 70, 71, 81, 101]. Nevertheless, there is still no single index that consistently outperforms the accuracy of PERCLOS in detecting drowsiness during driving. It will be necessary to gather and consolidate data from more studies to develop the best measure that accurately detects driving performance impairments related to drowsiness.

To overcome the issues of PERCLOS described above, integrating PERCLOS with other physiological or behavioral indicators has been attempted to provide a better estimation of a driver’s state or performance during driving, for example: (1) classifying drowsiness categories into three levels by integrating lane deviation of driving and PERCLOS [102, 103]; (2) estimating self-reported sleepiness during simulated driving in three levels (Karolinska Sleepiness Scale [KSS]: 1–6; KSS: 7; KSS: 8–9) by integrating PERCLOS, pupil diameter, SD of lateral position, and a steering wheel, with a multilevel ordered logit model [92]; or (3) models that predict lane-crossing or EEG-defined microsleeps by integrating eye metrics measured using Optalert (JDS scores, %TEC, and/or amplitude-velocity ratio), driving performance measures (SD of lane position, SD of steering wheel position, or steering wheel error), or driver effect [75]. Further validations of these methods under driving conditions are important in overcoming the current limitations of PERCLOS.

## Use of PERCLOS Under Aviation-related Conditions

The effectiveness of using PERCLOS for detecting drowsiness in aviation-related tasks is limited even though the task performance showed an effect of extended awake (Table 1) [64]. Additionally, there is an index that showed a higher correlation with the RT during the target acquisition task than that of PERCLOS (Table 2) [64]. Consequently, PERCLOS may not be a reliable technique for detecting performance deterioration caused by drowsiness during aviation-related tasks [64]. However, the same study did not show an increase in PERCLOS in the PVT associated with sleep deprivation, which has been shown repeatedly in previous studies [47, 51, 52]. The authors noted the high variability of PERCLOS is the cause of low sensitivity [64]. Further research will be required using a more stable technique to measure drowsiness-related performance impairment during aviation-related tasks.

## Use of PERCLOS for Sleep Detection in Patients With Sleep Disorders

Can PERCLOS be used to detect sleep onset in sleep disorders? A study determining whether PERCLOS would be a convenient method for detecting sleep during the MWT in patients with sleep disorders showed that PERCLOS had the best performance in discriminating the occurrence of sleep in an MWT compared to other metrics (Table 2) [55]. However, PERCLOS estimated sleep onset earlier than that defined by polysomnography (PSG), and the 95% confidence interval was 21.1 min. Therefore, Kratzel *et al*. [55]. proposed that PERCLOS alone cannot replace the method of detecting sleep during MWT in clinical settings. However, composite measures of PSG and eyelid closure (a component of PERCLOS) may improve the usefulness of MWT in clinical settings. In fact, such a combination successfully detects microsleep episodes in the MWT with a time resolution of 1 s [104]. Further development of this type of combination may improve the diagnosis, as well as the assessment of treatment and fitness-to-drive by in-depth analysis, of the wake–sleep transition zone.

## Other Applications of PERCLOS

### Time-on-task

Several studies have reported the effect of time-on-task on PERCLOS [51, 59, 72, 94, 105, 106]. The results revealed a time-on-task increase in PERCLOS during PVT, which is more pronounced from 20 to 34 h after awakening [51]. PERCLOS is also affected by time-on-task during driving simulator tasks [59] or on-road driving [72, 94]. The time-on-task effect is particularly pronounced at night in on-road driving [94], in individuals with multiple sclerosis in which daytime sleepiness is a common symptom [105], or during a task in which the observer is required additional effort to find a target appearing in one of the five locations [106]. PERCLOS is a sensitive index not only for drowsiness but also for time-on-tasks.

### Pre-driving alertness assessments

The utility of PERCLOS as a pre-driving alertness assessment has been investigated in a study that recorded naturalistic driving over 2 weeks in night shift workers with pre-driving alertness assessments, including ocular parameters and PVT before driving. The results revealed that the mean blink duration and PERCLOS were the best discriminators for predicting the occurrence of behavioral microsleeps, defined as an eye closure duration of >500 ms, during subsequent driving [107]. The results indicated the potential for the use of these eye indices for assessing fitness-to-drive.

### Prolonging effect on sleep time

Dinges *et al*. conducted a vigilance measurement study on professional truck drivers driving on actual roads [108]. The authors examined the effect of feedback from driving performance and vigilance measures such as lane tracking performance, PERCLOS, and PVT. Under conditions with feedback using these metrics, performance during nighttime driving improved, and non-workday sleep time after returning home increased. Vigilance detection systems not only inform people of their vigilance status but may also promote awareness of the importance of ensuring sleep [108].

### Hypoxia in cockpits

PERCLOS was recently identified as a potential biomarker for the early stages of hypoxia in cockpits [63]. PERCLOS responded with the fastest change during which SpO_2_ was gradually reduced by 5% in a console located inside a high-altitude hypobaric chamber among the indices tested. Thus, further studies should be conducted on the utility of PERCLOS as an index for the pilot’s state in aviation.

## Comparing PERCLOS and the JDS

The JDS [83, 109], like PERCLOS, is one of the most validated drowsiness indices in the field of sleep research. JDS scores increase with decreased performance during vigilance tasks [73, 74, 83, 84, 109] and simulated driving tasks [70, 83, 109–112]. Additionally, JDS scores were found to decrease with caffeine intake [110, 111], decrease with real-time drowsiness feedback [112], and increase with benzodiazepine use [81]. The increased JDS has also been observed during on-road driving after a night shift [93, 95] or extended wakefulness [113]. One of the advantages of the JDS is that, among drowsiness monitoring devices, validation in a variety of situations using a single device (i.e. Optalert system) is the most advanced for JDS compared with other measures [114]. In contrast, multiple devices are available for measuring PERCLOS, and validations with a single device are not as advanced compared with those for the JDS [114]. It should be noted that there are cases in which a single ocular metric was shown to be more accurate for detecting decreased PVT performance compared with the JDS [70, 74, 84].

Whether the JDS or PERCLOS is more accurate as a drowsiness measure is a research topic of interest [48, 70]. A study that simultaneously measured Copilot PERCLOS and Optalert JDS showed no difference in PERCLOS during PVT or driving tasks after sleep deprivation compared with those after normal sleep. However, there was a difference in JDS scores and %TEC showing increased values during both tasks after sleep deprivation compared with those after normal sleep [70]. The lack of difference in PERCLOS might be due to the limited capability of gaze direction and low sampling (i.e. 3 Hz) of the device [49]. Further research involving direct comparisons between the JDS and PERCLOS using simultaneous or consecutive measures within a single study is necessary.

## Comparing PERCLOS and Blink Duration

The increase in blink duration has been used to investigate the effects of sleep deprivation, partial sleep restriction, circadian misalignment, countermeasure of sleepiness and time-on-task on drowsiness in PVT [51], simulated driving [115, 116], on-road driving [72, 94, 117, 118]. A recent review suggested that blink duration and PERCLOS are the most frequently assessed parameters across studies among eyelid metrics measured using either an infrared sensor or EOG and consistently increased in response to extended wakefulness or low circadian alertness, regardless of the task type or acquisition technique, suggesting that blink duration and PERCLOS are robust measures of sleepiness [49].

The results of single studies comparing the accuracy of drowsiness detection using PERCLOS and blink duration are inconsistent among studies. While certain studies suggest that PERCLOS or %TEC are more accurate than blink duration in detecting drowsiness during the fixation [73], PVT [81], and OSLER test [58], other studies indicate that blink duration is more accurate than %TEC in detecting drowsiness during the auditory PVT [74] and OSLER test [84]. Additionally, some studies demonstrate that blink duration is more associated with crashes during simulated driving [59, 81], while others show that PERCLOS is more accurate in detecting line crossing [59]. Sleep detection during MWT is more accurate using PERCLOS than blink duration [55]. Detection of self-reported sleepiness is more accurate using %TEC in the fixation [73] and blink duration in the simulated driving [59]. More systematic comparisons of the accuracies between these indicators in various situations are necessary.

## Future Directions

As noted above, many studies have shown that drowsiness manipulation causes an increase in PERCLOS during several tasks (Table 1). However, cases in which PERCLOS is not affected by drowsiness manipulation have been reported especially in moderate drowsiness situations [69, 71, 91, 94] or less vulnerable populations to drowsiness [72, 91]. In addition, there is still little research regarding the usefulness of PERCLOS for detecting drowsiness in sleep disorders such as sleep apnea [55, 99] or aviation-related tasks [64]. To better understand the utility of PERCLOS, it will be necessary to accumulate data regarding which populations and situations the PERCLOS is effective in.

Many studies measuring PERCLOS during PVT and the OSLER test have reported that PERCLOS has the best accuracy among multiple measures [47, 52, 58]. However, studies that measured indices during driving [59, 68, 71, 81, 101] or aviation-related tasks [64] have reported that other indices exhibited superior performance for detecting drowsiness-related performance impairment compared with PERCLOS (Table 2). These findings may have resulted from the following technological limitations of PERCLOS: (1) inadequate detection of the eyes from the camera’s field of view because of head movement; (2) occlusion of the face because of glasses, sunglasses, etc.; or (3) inadequate measurement because of light reflection [52, 119]. To overcome these limitations, integrating several physiological and behavioral measures such as other eye and eyelid metrics [51, 120], HRV [52, 121], behavioral measures [68], or contextual information [122, 123] into PERCLOS may substitute for the evaluation of drowsiness when PERCLOS is not detectable, contribute to improving the detection accuracy of drowsiness-related performance deterioration by factors other than falling asleep, such as inattention or distraction [67, 72]. Integrating other indicators with PERCLOS may also contribute to improvements in two issues: (1) decreasing of accuracy as the evaluation time becomes shorter not only in PVT [47, 51] but also in driving tasks [71]; (2) false positives or false negatives in the detection of performance deterioration [52, 58, 67, 71].

Because of variations in the definition of PERCLOS and differences in sensing technology, different devices may output different PERCLOS values [71]. The results also differ when PERCLOS and %TEC are measured simultaneously [70]. Therefore, standardization of PERCLOS will be necessary for future research. In addition, many studies have used Optalert implementing JDS to conduct validation studies in various environments [114]. Validation using a single device that implements a PERCLOS-based method would facilitate comparisons between studies.

Although the development of real-time drowsiness measurements for drivers is particularly advanced, drowsiness is also a critical issue in a range of other professions, including flight crews [124], flight controllers [54, 125], astronauts [126–128], and medical personnel [129–131]. Validation of the application of drowsiness estimation in these professions may contribute to resolving drowsiness-related issues. For example, PERCLOS has recently been implemented in devices such as network cameras [132]. Such devices may enable early detection of drowsiness caused by work schedules [125, 129] or work type [54] by conducting periodic drowsiness measures of fixed-position staff [132] in offices, air traffic control units, and nurses' stations, in situations in which there is relatively little head movement, a minimal influence of ambient light, and no need to hide the face with sunglasses. For this purpose, the validation of drowsiness measurements during simulated tasks or real-world occupational scenarios is needed. The ability to unobtrusively and objectively assess drowsiness will increase opportunities for applying these measurements in the workplace, enable prompt countermeasures to drowsiness in the workplace, and prevent occupational human errors caused by drowsiness.

## Limitations

This review involved several limitations: first, because this was not a systematic review, the possibility of potential bias cannot be ruled out. In addition, although this review extracted related papers from several databases, including PubMed and Web of Science, some relevant studies may not have been included. Second, because the focus of the present review was to discuss PERCLOS, there may have been an insufficient discussion of drowsiness indices, such as blink duration or JDS, which are other well-validated passive measures of drowsiness. Third, the current review did not discuss the development of techniques or hardware for measuring PERCLOS with high levels of accuracy (e.g. computer vision, machine learning, or sensors).

## Conclusions

Many studies have shown that PERCLOS increases with sleep deprivation, after partial sleep restriction, at nighttime, and by other drowsiness manipulations during vigilance tests, simulated driving, and on-road driving. However, there are cases in which PERCLOS is not affected by drowsiness manipulation, such as in moderate drowsiness conditions, in older adults, and during aviation-related tasks. In addition, although PERCLOS is one of the most sensitive indices for detecting drowsiness-related performance impairments during PVT or the OSLER test, other indices exhibit superior performance compared with PERCLOS during simulated driving or aviation tasks. Another potential limitation of PERCLOS is that slight differences in the definition of PERCLOS and the presence of multiple measurement instruments may cause variations in PERCLOS values among different studies. Standardization of PERCLOS or validation across multiple studies using a single device to implement PERCLOS-based technology may be useful for clarifying the range of populations, tasks, and situations for which it is an effective measure. Furthermore, PERCLOS alone may not be sensitive for detecting drowsiness-related performance impairment by factors such as inattention or distraction other than falling asleep [67, 72]. The improvement of accuracy and validation studies of PERCLOS as a drowsiness measure by combining it with other indicators may be useful for overcoming its limitations. The development of PERCLOS-based technology will contribute to the prevention of human error and accidents caused by drowsiness in various occupational fields.

## Notes

This paper is part of the David F. Dinges Festschrift Collection. This collection is sponsored by Pulsar Informatics and the Department of Psychiatry in the Perelman School of Medicine at the University of Pennsylvania.
